# Fossil Elephant

**Published:** 1849-10

**Authors:** 


### FOSSIL ELEPHANT.

The remains of an elephant were lately exhumed in Vermont, and have been submitted to the inspection of Professor Agassiz, who states that it is the first true elephant found in a fossil state in the Northern States. The elephant is readily distinguished from the mastodon, found in so many parts of our country, by the shape of the teeth, from the form of which organs the latter animal takes its name.

